# Hospital and Health News

**Published:** 1923-07

**Authors:** 


### HOSPITAL AND HEALTH NEWS.

### Presentation to a Secretary.

Mr. C. J. Widdows, who has resigned the honorary secretaryship of Hitchin Hospital, has been presented with etchings by Howarth and Griggs. The cheque accompanying these gifts he has given to the hospital, of which he has become a life governor. Mr. Widdows will act jointly with Mr. W. T. Lucas as hon. treasurer.

### Ambulance Competition Tests.

Messrs. John Bale, Sons & Danielsson Ltd., publish three useful little folding leaflets on Ambulance Competition Tests (Stretcher, Individual and Question), by W. Corbet Fletcher, M.B., M.R.C.S. The tests are thoroughly up to date, and ambulance students, especially those in training for competitions, should find the leaflets of great help.

### "The Light of Healing."

To help the appeal for £150,000 on behalf of Wolverhampton General Hospital, a picture-paper called "The General's Despatch," illustrating every side of the hospital's work has been issued, price 3d. A lighthouse has also been built on the hospital's roof, which winks at night over an area of twenty miles. It represents the "light of healing," and cannot be missed.

### Braintree and Bocking Cottage Hospital.

Mr. Ernest Ingold, hon. treasurer of the new Braintree and Bocking Cottage Hospital, announces that the endowment fund amounts to £11,000. When Mr. W. J. Courtauld offered to present the building some three years ago, his great gift was doubtfully accepted for fear that it could not be maintained. The old hospitals subscriptions had long remained stationary.

### Mothercraft Posters.

Some effective Mothercraft Posters useful for display at Infant Welfare Centres, arranged by M. Olive Haydon, are published by Messrs. John Bale, Son & Danielson, Ltd. The set consists of 24, and the price per set is 7s. 6d. net The Posters are nicely got up and well calculated to catch the eye. The same firm publishes some sensible little Talks To Mothers, by Sister Lily Skene. late of Hammersmith Infant Welfare Centre. Series I is on "Infant Care," and series II. on "Character Training." Numbers one and two in each series are now ready, and the remainder will be issued monthly at the price of twopence.

### The late Mr. H. J. Boyd Carpenter.

The death of Mr. Henry John Boyd Carpenter, son of the well-known Bishop, at the age of fifty-eight, removes a man who, after a life devoted to education, became an ardent hospital worker. Through his efforts the Dartmouth and Kingswear Cottage Hospital was reopened, after it had apparently lapsed beyond recall, and, as its honorary treasurer, he worked hard for its endowment. Of the good work that goes on so quietly for our cottage hospitals, Mr. Boyd Carpenter's is a fine example, and it is through such men, with such motives as his, that the voluntary system is maintained.

### The Physiological Congress.

The eleventh International Physiological Congress will be held in Edinburgh, from July 23 to July 27. Those who desire to be enrolled as members are requested to forward their names and addresses, together with the amount of their subscription (25s.), to Miss Charlton, Physiology Department, University, Edinburgh, who will send on request particulars of hotels and lodgings, and all other necessary information. The expense of living in Edinburgh during the week of the congress will be from £3 upwards, according to the accommodation desired. Opportunities will be afforded for the exhibition of physiological apparatus.

### Hospital Cinematograph Film.

The authorities responsible for the organisation of the Hospital and Nursing Exhibition are prepared to lend the film illustrating the hospital work to any voluntary hospital in the provinces desiring to raise funds. It has been suggested that the film might be shown in the local cinematograph theatre on a Sunday by permission of the authorities, or any week-day by arrangement with the proprietor of the cinematograph theatre in question. Several photographs from this film were, it may be remembered, reproduced in our May issue. Applications for the film and terms should be made to the Secretary, Hospital, Nursing and Midwifery Exhibition and Conference, 22/24 Great Portland Street, W.1.

### Temporary Closing of Westminster Hospital.

Owing to the necessity of carrying out extensive repairs and alterations, Westminster Hospital will be closed for in-patients from July 2 for about six months. Out-patients will be treated at Westminster Hospital Medical School, 12 Caxton Street, but no casualty cases can be dealt with. Substantial improvements will be made in the wards and in the out-patient department, an additional operating theatre will be built, the X-ray, massage, and other departments will be brought thoroughly up to date, and better accommodation for the nurses will be provided in a home outside the hospital. The cost will be about £45,000, for which a special appeal will shortly be made.

### Congress of the Royal Sanitary Institute.

The thirty-fourth congress of the Royal Sanitary Institute will be held at Hull from July 30 to August 4. The Rt. Hon. T. R. Ferens, High Steward of Hull, is the president, and will deliver the inaugural address. There will be various sectional meetings and conferences, and a health exhibition illustrating municipal sanitation and domestic health and comfort will be held in the Wenlock Barracks. Nearly 200 authorities, including foreign and Dominion governments, government departments and local authorities have, up to the present, appointed delegates to the meeting. All further particulars can be obtained from the Offices of the Institute at 90, Buckingham Palace Road, S.W.1.

### The Safety Firelighter.

The "Certo" Patent Firelighter consists of a series of layers of materials joined together in such a way that when not in use it only measures 5 inches long by 1 inch thick by 1 inch wide; but when spread out ready for use it attains a circumference of 18 inches. When not in use it readily adapts itself for packing, handling, transporting and storing. A parcel containing 100 only measures about 6 inches by 6 inches by 10 inches, and therefore a very large quantity can be stocked in a very small space. When ready for use the firelighter is large enough and strong enough to bear the weight of a grate of coal, and is so transformed as to incorporate air spaces, without which it would be impossible to light any fire successfully. Given decent coal and a fair upward draught a "Certo" will ignite any ordinary fire, it will save money, and economise time, and is altogether a useful addition to every household. "Certo" is the only firelighter used by the Government.

### Commemoration at Livingstone College.

Speaking at a successful Commemoration Day at Livingstone College, Sir Leonard Rogers, to whom the world owes so much for his wonderful discoveries in the treatment of dysentery, cholera, kala-azar and leprosy, referred to the branch of medicine in which he is greatly interested, and in which he feels the very great aid of missionaries will be required if full use is to be made of the recent discoveries—the great problem of dealing with leprosy. Sir Leonard pointed out that it is impossible to carry on with the present supply of workers, and he felt strongly that if those missionaries who were going out to work amongst lepers were sent to be trained at Livingstone College it would be very beneficial in helping to carry out the cure of leprosy. He spoke of the problem of segregation and prophylaxis, and the difficulties in carrying out these measures. "We shall have to look to the missionaries to help us," he said, "in this work, and we shall have to send them to this college before they go out. When that can be done we shall make an enormous advance."

